# Exuberant pyogenic granuloma in extragingival site

**DOI:** 10.1590/S1808-86942012000400025

**Published:** 2015-10-20

**Authors:** Fábio Wildson Gurgel Costa, Ana Thayssa Tomaz Lima, Roberta Barroso Cavalcante, Karuza Maria Alves Pereira

**Affiliations:** 1 PhD student of dentistry - UFC (Assistant Professor I - UFC Campus Sobral Dentistry Program); 2 Professor of Dentistry - UFC Campus Sobral; 3 PhD in Oral Pathology - UFRN (Professor of Dentistry - University of Fortaleza (UNIFOR)); 4 PhD in Oral Pathology - UFRN (Adjunct Professor of Dentistry - UFC Campus Sobral)

**Keywords:** granuloma, mouth, pyogenic, tongue

## INTRODUCTION

Non-neoplastic proliferative processes are a group of disorders relatively common in the oral cavity. Among them we stress the pyogenic granuloma (PG) because of its occurrence, typical classical characteristic presentation and distribution in gingival sites in over 80% of the cases^1^, ^2^, ^3^, ^4^, ^5^, ^6^, ^7^. Nonetheless, when extragingival lesions appear, a rare condition listed in the literature, diagnosis may be late^1^, ^2^, ^3^, ^4^, ^5^, ^6^, ^7^. Thus, this paper aims at reporting a case of exuberant pyogenic granuloma in a non-gingival site.

## CASE REPORT

**Figure 1:**
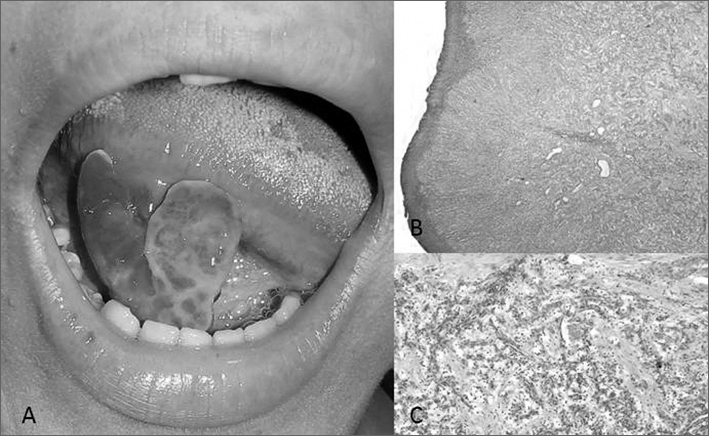
A: Clinical view of the exuberant lesion on the tongue B-C: Microphotography showing thin para-keratinized stratified squamous epithelium with numerous blood vessels (Hematoxylineosin, 200x).

16-year old female black patient came to the stomatology ward of the University Hospital at the Federal University of Ceará, ***Campus*** Sobral, complaining of a “growth in her tongue” with pain, bleeding upon touch, perceived two months after an accident. Upon intraoral physical exam (Figure 1A), we spotted a reddish exophytic lesion was seen, with an ulcerated surface, bleeding upon minimum manipulation, soft, measuring approximately 3.0cm in its longest diameter, and located in the posterior region of the right tongue border. Upon extraoral exam, nothing important was noticed. Having that, the main diagnostic considered was that of a pyogenic granuloma. Considering the medical interview and the clinical findings, treatment was based on complete lesion exeresis. In an outpatient ward and under local anesthesia, the material was harvested and sent to histopathology, which matched clinical findings. During surgery, clinical maneuvers were used to minimize bleeding, as a good anesthesia on the basis of the lesion and continuous suture. Upon microscopic examination (Figure 1B-C), they found a thin para-keratinized stratified squamous epithelium with connective tissue, with numerous blood vessels of varied gauges, and these areas were, sometimes, interspersed by inflammatory cells. The patient has been under clinical follow up for 8 months, without signs of recurrence.

## DISCUSSION

Granuloma pyogenic development is usually associated with an exuberant response to chronic irritative local factors of low intensity, or trauma, with hormonal factors impacting and, therefore, very often found among pregnant women. It is more prevalent among women in their second to fourth decades of life, and it may involve the skin, lips, tongue, cheek mucosa and palate, being more common in the anterior teeth gingiva.

We carefully reviewed the literature^1^, ^2^, ^3^, ^4^, ^5^, ^6^, ^7^ with comparative purposes, taking as bases the main series of cases published by 2010, making up a total of 1,127 cases and, among these, there were only 79 on the tongue - 7% of the cases. As far as the etiology is concerned, it has been reported that 80% of the patients with extragingival oral pyogenic granuloma responded positively on local injuries where the lesions appeared. The main age range was within the second and third decades of life and females were the most affected in 100% of the cases studied - which corroborated the case hereby reported. The lesion exeresis, as per carried out in the present case, is according to the literature, the best treatment approach for these pyogenic granulomas.

Regarding the low occurrence of PG in extragingival sites, it is pertaining to emphasize the importance of the correct diagnosis of these lesions, distinguishing them from other entities which have similar characteristics, so as to obtain a proper treatment approach. In this paper, we stress the importance of professionals who work with the oral cavity, notably dentists and otorhinolaryngologists, in recognizing hyperplastic reactive lesions, even when located in unusual sites, aiming at an early treatment, without harming the patient.
